# The immunology of Zika Virus

**DOI:** 10.12688/f1000research.12271.1

**Published:** 2018-02-19

**Authors:** Abigail Culshaw, Juthathip Mongkolsapaya, Gavin Screaton

**Affiliations:** 1 Department of Medicine, Imperial College London, Hammersmith Campus, Du Cane Road, London, W12 0NN, UK; 2 Dengue Hemorrhagic Fever Research Unit, Office for Research and Development, Siriraj Hospital, Faculty of Medicine, Mahidol University, Bangkok, Thailand; 3 Medical Sciences Division, University of Oxford, Level 3, John Radcliffe Hospital, Oxford, OX3 9DU, UK

**Keywords:** Zika virus, dengue virus, innate immunity, antibodies, T cells, immunopathogenesis, vaccine

## Abstract

Zika virus (ZIKV) was initially thought to cause only mild, self-limiting symptoms. However, recent outbreaks have been associated with the autoimmune disease Guillain-Barré syndrome and causally linked to a congenital malformation known as microcephaly. This has led to an urgent need for a safe and effective vaccine. A comprehensive understanding of the immunology of ZIKV infection is required to aid in the design of such a vaccine. Whilst details of both innate and adaptive immune responses to ZIKV are emerging, further research is needed. As immunopathogenesis has been implicated in poor outcomes following infection with the related dengue virus, identification of cross-reactive immune responses between flaviviruses and the impact they may have on disease progression is also of high importance.

## Introduction

**Figure 1.**
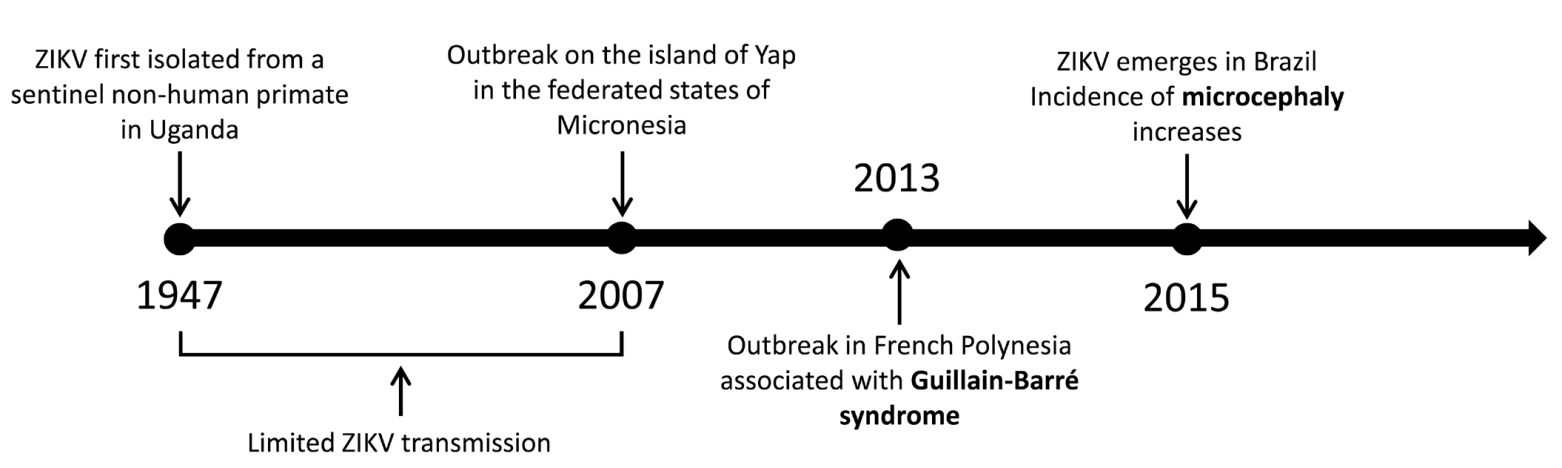
Timeline of the spread of Zika virus (ZIKV). Following its isolation in Uganda in 1947, ZIKV caused only sporadic incidences of human infection until an outbreak on the island of Yap in 2007. Since then, the virus has spread through the South Pacific to South America, resulting in large-scale transmission that is associated with severe complications such as Guillain-Barré syndrome and microcephaly.

Zika virus (ZIKV) was first isolated from a febrile non-human primate in the Zika Forest of Uganda in 1947 ^1^ ( Figure 1). Very few cases of human infection were reported in the subsequent decades ^2^ until 2007, when an outbreak occurred on the island of Yap in the Federated States of Micronesia ^3^. In 2013, some 30,000 cases of ZIKV infection were recorded in French Polynesia ^4^. Up to this point, the virus was believed to cause only a mild, self-limiting febrile illness. However, ZIKV transmission in French Polynesia was associated with the development of the neurological autoimmune disease Guillain-Barré syndrome (GBS) ^5^.

After 2013, ZIKV emerged in South America, first appearing in Chile ^6^ and then going on to cause a large-scale outbreak in Brazil. It is estimated that by the end of 2015 between 440,000 and 1.3 million people had been infected in Brazil ^7^. This increase in ZIKV prevalence was coincident with a rise in the occurrence of a congenital malformation known as microcephaly ^8^. This birth defect results in a reduction in head size, causing several complications, including developmental delay.

Owing to the severe nature of complications arising from ZIKV transmission, the development of a safe and effective vaccine has become a priority for the research community. An extensive understanding of the immunology of ZIKV infection is vital to inform the design of such a vaccine. Owing to the relatedness of ZIKV to other flaviviruses that co-circulate in endemic regions, it is also of importance to understand how cross-reactive immune responses might impact on the outcome of ZIKV infection.

## Zika virus

ZIKV is a member of the flavivirus family, which includes other important human pathogens such as dengue virus (DENV), yellow fever virus (YFV), West Nile virus (WNV), Japanese encephalitis virus, and tick-borne encephalitis virus. ZIKV is an arthropod-borne virus that is transmitted via the bite of infected *aedes* mosquitoes ^9^. Seemingly unique amongst flaviviruses, ZIKV can also be transmitted by the aforementioned perinatal route as well as via sexual contact ^10, 11^. Furthermore, the sexual transmission of ZIKV can potentially occur long after acute infection, as virus has been detected in semen more than 6 months after symptomatic disease ^12^. ZIKV has also been shown to cause damage to the testes and affect fertility in a mouse model of infection ^13^.

As with other flaviviruses, ZIKV has a single-stranded positive-sense RNA genome that is translated into a single polyprotein. This polyprotein is cleaved into three structural proteins—capsid, pre-membrane (PrM), and envelope (E)—and seven non-structural (NS) proteins: NS1, NS2a, NS2b, NS3, NS4a, NS4b, and NS5. The surface structure of the mature ZIKV virion is composed of 90 anti-parallel E dimers arranged in a herringbone formation ^14^. This is similar to the structure of both DENV and WNV.

## Immune responses to Zika virus

### The innate immune response

The interferon (IFN) response plays a critical role in the control of flaviviruses, as shown by the increased susceptibility of mice lacking components of the IFN pathway to flaviviral infection ^15, 16^ and the numerous mechanisms employed by flaviviruses to counteract this control ^17, 18^.

Several *in vitro* studies using both primary human cells and human-derived cell lines have been undertaken to assess the IFN response to ZIKV infection. Dependent on the cell type used, ZIKV infection resulted in the production of type I (α, β), type II (γ), and type III (λ) IFN as well as the activation of several IFN-stimulated genes (ISGs) ^19– 22^.

Investigations to determine the mechanisms employed by ZIKV to dampen the IFN response have also been carried out. The ZIKV NS5 protein has been shown to degrade STAT2, a signalling molecule involved in the type I IFN pathway ^23^. This limits type I IFN signalling and allows increased viral replication. This appears to be a species-specific interaction, as ZIKV NS5 is unable to degrade murine STAT2, contributing to the inefficient replication of ZIKV seen in immunocompetent mice and the increased susceptibility of mice lacking components of the type I IFN signalling pathway to infection with ZIKV.

However, it should be noted that this study relies on the over-expression of NS5. Furthermore, another investigation using infected human dendritic cells suggests another mechanism of ZIKV-mediated IFN inhibition. That study found ZIKV infection to result in the antagonism of STAT1 and STAT2 phosphorylation ^24^. As yet, it is unclear which mechanism might occur first or whether both take place during natural infection.

### The antibody response

The structural proteins E and PrM along with the secreted protein NS1 represent the major targets for flavivirus-specific antibodies ^25, 26^. Initial studies show that the ZIKV-specific antibody response conforms to this pattern ^27^, although additional comprehensive mapping experiments would be beneficial.

Studies have shown ZIKV-specific antibodies to be crucial for viral control in mouse models of infection. A DNA vaccine comprising ZIKV PrM and E protected mice from viral challenge, resulting in an absence of detectable viremia. This protection was mediated by E-specific antibodies, as demonstrated by passive transfer experiments ^28^. This same vaccine was also found to be protective in rhesus macaques ^29^.

Several human monoclonal antibodies capable of neutralising ZIKV both *in vitro* and *in vivo* have been isolated. These include antibodies to the quaternary E dimer epitope, which cross-react between ZIKV and DENV ^30– 32^. Antibodies recognising domain III of ZIKV E protein, which reduce disease symptoms and mortality in ZIKV-infected mice, have also been identified ^33^. In addition, two antibodies, which recognise epitopes spanning multiple domains of the ZIKV E protein, were found to protect mice in a post-exposure model ^34^.

### The T-cell response

The immunodominant DENV-specific CD8 ^+^ T-cell response is directed toward the NS proteins NS3 and NS5, although epitopes have been identified across the genome. Further work is required to determine which ZIKV proteins are the major targets of the T-cell response in humans.

One study in IFN receptor (IFNR) type I-deficient mice found ZIKV-specific CD8 ^+^ T cells to predominantly target the PrM, E, and NS5 proteins. It was demonstrated that such cells have a protective role during infection, as adoptive transfer led to a reduction in virus load following challenge ^35^. In an IFNR type I-deficient HLA transgenic mouse model, ZIKV-specific T cells were found to recognise epitopes across all 10 proteins. These T cells were also shown to be protective during ZIKV infection, as immunisation with peptide epitopes resulted in a reduction in virus load following challenge ^36^. This reduction was abrogated following the depletion of CD8 ^+^ T cells.

Depletion of T cells resulted in ZIKV-induced weight loss in both wild-type and IFN-deficient mice. This study also found CD4 ^+^ and CD8 ^+^ T-cell responses toward ZIKV to be dampened during pregnancy ^37^. If this is the case for human ZIKV infections, it could have implications for the development of microcephaly.

Caution is required when extrapolating data from mouse studies to be applied to humans. With the exception of the HLA transgenic model, the mice used in the experiments described above display distinct major histocompatibility complex molecules to humans. This will lead to the recognition of different T-cell epitopes. In addition, many of these studies were carried out in mice lacking components of the IFN pathway, which plays an important role in the generation of a fully functional T-cell response.

Studies investigating T-cell responses during human ZIKV infection are sparse at present. CD4 ^+^ T cells that proliferate in response to stimulation with E and NS1 have been identified in humans who had experienced a recent ZIKV infection ^27^, and *in silico* analysis has been used to predict ZIKV T-cell epitopes ^38^. Therefore, substantial further research is required to confirm a protective role for T cells during ZIKV infection in humans.

## Flavivirus cross-reactive immune responses: immunopathogenesis or protection?

ZIKV transmission is taking place in areas where other flaviviruses are circulating. Therefore, ZIKV infections will be occurring in populations with previous flavivirus immunity. It will be of importance to understand how such immunity will impact on the outcome of ZIKV infection. Particular focus has been given to cross-reactivity between DENV and ZIKV, as the E proteins are closely related ^30^. Moreover, immunopathogenesis has been implicated in contributing to the severe symptoms of DENV infection.

DENV co-circulates as four closely related serotypes, and sequential infections are common. Individuals experiencing a secondary or subsequent infection are more likely to develop serious complications ^39, 40^, suggesting a role for virus-specific immunity in pathogenesis. The mechanism for antibody involvement is well defined. Subneutralising levels of DENV-specific antibodies can lead to opsonisation of virus particles and increased entry into Fc receptor-bearing cells ^41^. Such cells are a major site of DENV replication, thus leading to increased viral replication in a phenomenon known as antibody-dependent enhancement (ADE).

The role of serotype cross-reactive T cells in DENV pathogenesis is more contentious. CD8 ^+^ T cells that preferentially recognise epitopes derived from the primary infecting serotype have been identified ^42^. The expansion of cells generated during a primary infection but displaying a low avidity for the current infecting serotype may result in delayed viral clearance and higher viral loads. However, studies in both humans and mice have suggested a protective role for serotype cross-reactive T cells ^43– 46^.

The role that immunopathogenesis plays in the outcome of DENV infection along with the genetic similarities and co-circulation of both ZIKV and DENV lead to some important questions that need addressing. Can DENV-specific immune responses affect the outcome of ZIKV infection and vice versa? How might pre-existing flaviviral immunity impact on the maternal–fetal transmission of ZIKV? What implications might the answers to these questions have for vaccine design?

### Dengue virus cross-reactive antibodies

Several studies have identified antibodies that are cross-reactive between DENV and ZIKV ^27, 30– 33, 47, 48^. Furthermore, it has been demonstrated that DENV-specific antibodies are able to enhance infection of Fc receptor-bearing cells with ZIKV *in vitro* ^31, 47, 49, 50^. Adoptive transfer of DENV convalescent plasma enhanced pathogenesis in a mouse model of ZIKV infection ^49^, suggesting a role for ADE *in vivo*. Conversely, pre-existing DENV immunity did not result in more severe ZIKV disease in rhesus macaques ^51^.

However, both of these studies have limitations that should be considered when interpreting their data. The adoptive transfer experiments used immunocompromised mice, and the macaques displayed no symptoms of disease following infection. In addition, differences in experimental design may account for the disparate findings seen. Whilst antibodies alone were transferred into the mice two hours prior to infection, the macaques were infected with DENV two and a half years before ZIKV exposure. This may impact on the nature of the DENV-specific antibody response present. Moreover, in the macaques, other components of a DENV-specific immune response would be present alongside the antibodies, potentially affecting the outcome of ZIKV infection.

Therefore, whether or not the mechanism of ADE is at play during natural infection requires further research, and in particular the development of serological reagents to distinguish previous ZIKV and DENV infections is essential to probe epidemiological cohorts. DENV-specific antibodies with the ability to neutralise ZIKV infection *in vitro* have been identified ^27, 30– 32^, and monoclonal antibodies isolated from patients with dengue have been found to protect mice during ZIKV challenge ^32, 33^.

Of importance to this mechanism is the question of which cell types provide the major site of replication for ZIKV. If ZIKV predominantly replicates in non-Fc receptor-bearing cells, then DENV-specific antibodies are unlikely to have a major impact on pathogenesis. There is mounting evidence to suggest that the tissue tropism of ZIKV is distinct from that of DENV ^52^.

It may be that certain serotypes of DENV differ in their ability to generate a pathogenic or a beneficial antibody response. It may also be of importance how many previous DENV infections an individual has been exposed to. One previous DENV infection may enhance Zika disease, whereas two or more may turn out to be protective, as is the case for sequential DENV infections ^53^. As is also the case for DENV, the time gap between infections could be crucial ^54^.

### Dengue virus cross-reactive T cells

In addition to antibodies, DENV and ZIKV cross-reactive T cells have been identified. The study described above involving humanised mice identified T cells specific for several epitopes that were either ZIKV specific or cross-reactive between ZIKV and DENV. Both types of peptide epitope were used to immunise mice prior to ZIKV challenge and were found to be protective ^36^. Although the peptides used in this study were divided into two groups based on their cross-reactivity to DENV, all were exactly sequence-matched to the strain of ZIKV used to challenge the mice. To develop these findings further, it would be useful to ascertain whether T cells specific for DENV that are partially cross-reactive toward ZIKV are also protective.

In another mouse model of infection, CD8 ^+^ T cells from ZIKV-infected wild-type mice were found to protect type I IFNR knockout mice during both ZIKV and DENV infection ^55^. It has also been shown that adoptive transfer of DENV-specific CD8 ^+^ T cells can confer protection during ZIKV infection of type I IFNR knockout mice ^56^.

The previously mentioned study that found memory T cells specific for NS1 and E following ZIKV infection of humans demonstrated these cells to be poorly cross-reactive with DENV, even in those individuals who had experienced a previous DENV infection ^27^. In contrast, *in silico* analysis has identified T-cell reactivity conserved across several flaviviruses, including ZIKV predominantly found in the NS3 and NS5 proteins ^38^. In addition, a study of a human cohort revealed previous DENV exposure to affect the magnitude, timing, and quality of the ZIKV-specific T-cell response ^57^. Substantial further research is required to establish the role that T cells play during ZIKV infections and whether or not DENV cross-reactive T cells can contribute to pathogenesis or protection.

## Vaccine development

Several vaccine strategies have been found to be protective in either mouse or non-human primate models of ZIKV infection. These include DNA vaccines ^28, 29, 58, 59^, RNA vaccines ^60, 61^, live attenuated virus ^62^, inactivated virus ^28, 29, 63^, and virus-like particles ^64^. Vaccines have also been found to block the transmission of ZIKV to the foetus of pregnant mice ^65^. Some of these vaccines are currently in the early stages of clinical trials in humans ( Table 1).

**Table 1.** Current Zika virus vaccine trials.

Phase	Name	Strain	Target	Strategy	Sponsor	References
I	VRC-ZKADNA085-00-VP	H/PF/2013	PrM-E	DNA vaccine	National Institutes of Health	28, 29
	VRC-ZKADNA090-00-VP	H/PF/2013	PrM-E	DNA vaccine	National Institutes of Health	
	GLS-5700	Consensus	PrM-E	DNA vaccine	GeneOne	59
	ZPIV	PRVABC59	Whole virion	Inactivated virus	Walter Reed Army Institute of Research/National Institute of Allergy and Infectious Diseases	28, 29
	MV-ZIKA		E	Viral vector	Themis	
I/II	mRNA-1325	Micronesia 2007	PrM-E	mRNA vaccine	Moderna Therapeutics	61

E, envelope; PrM, pre-membrane.

Most of these vaccines are aimed at generating antibodies specific for the ZIKV virion. Analysis of other flaviviral vaccines has shown levels of E-specific antibodies to correlate with protection in both animals and humans ^66^. However, it has been suggested that the lack of efficacy of the recently licensed DENV vaccine may be due in part to its inability to generate an effective DENV-specific T-cell response, as the NS proteins contained within it are derived from YFV ^67^. Therefore, developing a ZIKV vaccine that aims to induce both an antibody and a T-cell response may prove beneficial.

A ZIKV vaccine may need to be used in pregnant women or immunocompromised individuals, which could raise safety concerns with live attenuated vaccines because of the risk of reversion. Such vaccines are, however, cost-effective to produce and often result in durable immunity, even after one dose. Other vaccine strategies present viable alternatives to live attenuated vaccines. DNA plasmid vaccines are easy to produce and are physically and genetically stable, and inactivated vaccines are already in use for other flaviviruses. However, both of these vaccine strategies may turn out to be less immunogenic as compared with a live vaccine ^66^.

Another consideration during the development of a ZIKV vaccine is that it may be difficult to determine the threshold of protection to prevent foetal transmission and microcephaly. Complete sterilising immunity may be required to prevent infection of the foetus, and assessing whether this has been achieved during a vaccine trial in which the endpoint is prevention of symptomatic infection will be challenging.

Monitoring should be undertaken to ensure that vaccination does not lead to increased susceptibility to severe outcomes following infection with other flaviviruses such as DENV. The mechanism of GBS following ZIKV infection, which occurs in an estimated 1 in every 4,000 cases ^5^, is not yet clear. However, auto-reactive antibodies have been shown to be present during GBS triggered by other pathogens ^68^. It will therefore be important to monitor vaccinees for symptoms of GBS.

## Conclusions

Since the association between ZIKV and severe complications such as GBS and microcephaly emerged, much research has been carried out, resulting in a substantial increase in our knowledge of the immunology of ZIKV infection. Proteins and, in some instances, epitopes targeted by antibodies and T cells during human ZIKV infection have been determined. Following the development of mouse models, protective antibody and T-cell responses have been identified. Several vaccine strategies have been tested in animal models, and early clinical trials have begun in humans. Caution and further insight are required in order to ensure that such vaccines will have no detrimental effects and will provide long-term protection.

Immunopathogenesis has been implicated as a contributing factor to DENV disease. Sequential infections with DENV and ZIKV are likely to occur because of the co-circulation of both viruses in endemic areas. DENV/ZIKV cross-reactive immune responses have already been identified, which is perhaps unsurprising given the sequence homology between the two viruses. A DENV vaccine is currently licensed for use in several countries experiencing ZIKV outbreaks. Gaining a comprehensive understanding of the effect that DENV immunity has during ZIKV infection and vice versa is crucial for the design of safe vaccine strategies.

## Abbreviations

ADE, antibody-dependent enhancement; DENV, dengue virus; E, envelope; GBS, Guillain-Barré syndrome; IFN, interferon; IFNR, interferon receptor; NS, non-structural; PrM, pre-membrane; WNV, West Nile virus; YFV, yellow fever virus; ZIKV, Zika virus.
